# The effect of sleep deprivation on lens-induced-myopia in guinea pigs

**DOI:** 10.1186/s42826-026-00285-7

**Published:** 2026-06-25

**Authors:** Yao Chen, Xueli Yang, Junfeng Mao

**Affiliations:** 1 Department of Ophthalmology, Xiangya Hospital, Central South University, Changsha, China; 2 Hunan Key Laboratory of Ophthalmology, Xiangya Hospital, Central South University, Changsha, China; 3 National Clinical Research Center for Geriatric Disorders, Xiangya Hospital, Central South University, Chang Sha, China; 4 Eye Center of Xiangya Hospital, Central South University, Changsha, Hunan Province 410008 PR China

**Keywords:** Sleep deprivation, Sigma-1 receptor, Myopia, Guinea pig, Glutamic acid

## Abstract

**Background:**

Myopia is one of the most common refractive vision disorders. Recent studies have demonstrated that insufficient sleep correlates with myopia development in adolescents. However, the underlying mechanism for myopia progression is unclear. This study aimed to investigate the effect of sleep deprivation (SD) on the myopia development of guinea pigs.

**Results:**

The experiment was divided into two parts. In the initial part, 45 animals were evenly divided into three groups, namely, the LIM(lens-induced myopia) group, LIM+SD20hr group, and LIM+SD21hr group. Thirty-six animals were used in the second part and divided into three groups: the LIM+SD20hr, LIM+SD20hr + NE-100 (Sigma-1 receptor antagonist), and LIM+SD20hr+saline groups. For animals of the LIM+SD20hr group, apart from wearing a concave lens on the right eye, they were deprived of sleep for 20 h daily. The LIM+SD21hr group was deprived of sleep for 21 h daily instead. For the LIM+SD20hr + NE-100 group, peribulbar injections of NE-100 60 µg were administered in the right eye. The LIM+SD20hr+saline group was administered with saline instead. The expression of retinal Sigma1R was measured using immunofluorescence staining and a Western blot. γ-aminobutyric acid (GABA) and glutamic acid (Glu) content in the retina was determined using the high-performance liquid chromatography electrochemical method. In the first set of experiments, SD alone did not induce myopia in control eyes but accelerated LIM progression, with increased myopic refraction (-3.14 ± 0.19 D vs. -1.93 ± 0.16 D in LIM alone, p < 0.001), axial elongation (8.34 ± 0.02 mm vs. 8.21 ± 0.01 mm, p < 0.01), and elevated retinal Glu/GABA ratio (p < 0.001). S1R expression was upregulated in LIM + SD eyes (p < 0.001). In the second set of experiments, intravitreal NE-100 administration partially reversed these effects, reducing myopic shift (-1.86 ± 0.20 D, vs. -3.21 ± 0.21 D In LIM + SD) and normalizing Glu/GABA levels (p < 0.001).

**Conclusions:**

SD exacerbates LIM via S1R activation and neurotransmitter dysregulation. S1R antagonists like NE-100 may offer therapeutic potential for myopia control.

**Supplementary Information:**

The online version contains supplementary material available at 10.1186/s42826-026-00285-7.

## Background

Myopia is among the most prevalent refractive vision disorders and can potentially lead to blindness in adulthood [1]. In East and Southeast Asia, the prevalence of myopia among young adults is alarmingly high, with 80–90% affected, and there is a significant incidence of high myopia [2]. High myopia is associated with an increased risk of severe complications, including cataracts, glaucoma [3], maculopathy, retinal detachment, and even blindness [4]. Mitigating the onset of myopia and curbing the progression of high myopia in school-aged children and young adults could alleviate the personal, societal, and economic burdens associated with these complications [5]. Various interventions have been proposed to manage its progression, including low-dose atropine [6], peripheral defocus modifying contact lenses [7], and orthokeratology [8]. However, the effectiveness of these interventions is constrained by adverse drug reactions [9], individual compliance, and educational pressures [2].

Retina-regulated scleral remodeling has been established as a contributing factor to myopia in animal studies. Interventions such as eyelid suturing, the use of eye shields, and the application of minus lenses are employed to alter the visual signals received by the retina. This alteration subsequently influences the biosynthesis and secretion of myopia-related signaling molecules, including dopamine (DA), nitric oxide (NO), retinoic acid (RA), vasoactive intestinal peptide (VIP), glutamic acid (Glu), and γ-aminobutyric acid (GABA). These signaling molecules activate subsequent pathways in retinal pigment epithelial cells, leading to scleral remodeling and an increase in axial length, thereby contributing to the development of myopia [10, 11]. Recent research has indicated a correlation between insufficient sleep and the progression of myopia in adolescents, with findings suggesting that adolescents and young adults who experience reduced sleep duration are at a higher risk of developing myopia [1, 12]. Furthermore, sleep deprivation (SD) in animal models has been shown to downregulate Glu [13], Glu transporter 1 [14], dopamine D2 receptor (D2R) [15], and upregulate DA [16] and GABA [17]. We hypothesize that SD may have implications for the development of myopia; however, the exact mechanisms by which sleep deprivation induces myopia remain to be elucidated.

In recent years, the Sigma-1 receptor (S1R) has garnered significant attention as a therapeutic target for neurodegenerative diseases. S1R functions as a molecular chaperone within cells, particularly in mitochondria-associated endoplasmic reticulum membranes [18]. These include calcium (Ca2+) homeostasis, the endoplasmic reticulum stress response [19], oxidative stress response, neurotrophic factor secretion [20], and glial cell activation [21], all of which contribute to its neuroprotective role in neurodegenerative conditions [22]. Activation of S1R in the brain modulates signaling pathways mediated by DA, NO, Glu, and GABA through the formation of heterodimers with the D2R, thereby attenuating the DA pathway [23]. Additionally, activated S1R interacts with the dopamine transporter (DAT), resulting in elevated extracellular DA concentrations by inhibiting the reuptake of DA from the synaptic cleft [24]. Moreover, S1R activation leads to increased extracellular Glu levels by inhibiting the Glu transporter 1 [25]. In S1R knockout mice, reductions in NO and neuronal nitric oxide synthase (nNOS) are observed, which subsequently suppress GABA neurotransmission [26]. Given the pivotal roles of signaling factors such as GABA, Glu, and DA in the development of myopia [11], it is hypothesized that S1R may also influence the regulation of myopia. Our previous research demonstrated that a S1R antagonist could inhibit form-deprived myopia in guinea pigs, an effect associated with increased dopamine levels in the retina [27].

This study seeks to ascertain whether sleep deprivation impacts myopia in a guinea pig lens-induced myopia (LIM) model by examining optic parameters and myopia-related signaling pathways involving Glu and GABA. Notably, S1R was activated during sleep deprivation, and the myopia induced by sleep deprivation could be mitigated by the S1R antagonist NE-100.

## Methods

### The aim, design and setting of the study

This study aimed to investigate the role of the S1R in SD-promoted myopia progression in LIM guinea pigs and to evaluate the potential therapeutic effect of the S1R antagonist NE-100. A prospective, randomized, controlled animal experiment was conducted. Eighty-one male tricolored guinea pigs, aged 21 days and weighing between 140 and 170 g, were utilized in the experiments. These animals were supplied by the Laboratory Animal Department of Central South University. All experimental procedures involving animals received approval from the Medical Ethics Committee of Xiangya Hospital, affiliated with Central South University, under protocol number 2,019,030,405. The care and use of the experimental animals complied with the “Regulations on Management of Experimental Animals” issued by the State Scientific and Technological Commission. Furthermore, the experiments were conducted in accordance with the ethical principles outlined in the guidelines of the Association for Research in Vision and Ophthalmology.

### Main reagents and instruments

The rabbit anti-S1R polyclonal antibody (ab151288) and the mouse anti-GAPDH polyclonal antibody (ab181603) were procured from Abcam, Cambridge, United Kingdom. The S1R antagonist, N,N-diethyl-2-[4-methoxy-3-(2-phenylethoxy) phenyl] ethanamine hydrochloride (NE-100), was sourced from Sigma-Aldrich, United States (SML0631). Immunohistochemistry was conducted utilizing the Polink-2 Plus Polymer HRP Detection System (PV-0023) from Beijing Biosynthesis Biotechnology Co., Ltd, Beijing, China. A bicinchoninic acid protein quantification kit (c05-02001) was also acquired from Beijing Biosynthesis Biotechnology Co., Ltd. The PVDF membrane (IPVH00010) and the Immobilon Western Chemiluminescent HRP Substrate (RPN2232) were obtained from Millipore. The prestained protein marker (26617) was sourced from Thermo. Additionally, the Waters 2695 high-performance liquid chromatograph and the Waters µBondapak C18 chromatographic column were acquired from Waters, United States. The − 6 D lenses were sourced from Hong Kong Optical Lens Co., Ltd., located in Hong Kong, China. The computerized optometry unit (PARK 1) was procured from OCULUS in Arlington, Germany. The keratometer (ARK-510 A) was acquired from Nidek, based in Aichi, Japan. The Aicevoos digital illuminometer (V8) was obtained from Zhongce Hongtu Measuring Instrument Co., Ltd., in Wuhan, China. The Aosvi binocular continuous zoom stereoscope (XTL-2600) was sourced from Aosvi Optical Instrument Co., Ltd., situated in Shenzhen, China. Axial length measurements were conducted utilizing an ophthalmic A/B ultrasound device procured from Tianjin Meda Co., Ltd., Tianjin, China (Model: ODM-2100 S; software version: 21SAB V1.20), featuring a resolution of 0.01 mm. The probe was manually aligned under anesthesia, with the animals being gently restrained to minimize motion artifacts. Intra-observer reproducibility was verified by calculating the coefficient of variation across three repeated measurements per eye, resulting in coefficient of variation values of less than 5% for all parameters.

### Lens-induced myopia (LIM) and sleep deprivation (SD) in guinea pigs

**Fig. 1:**
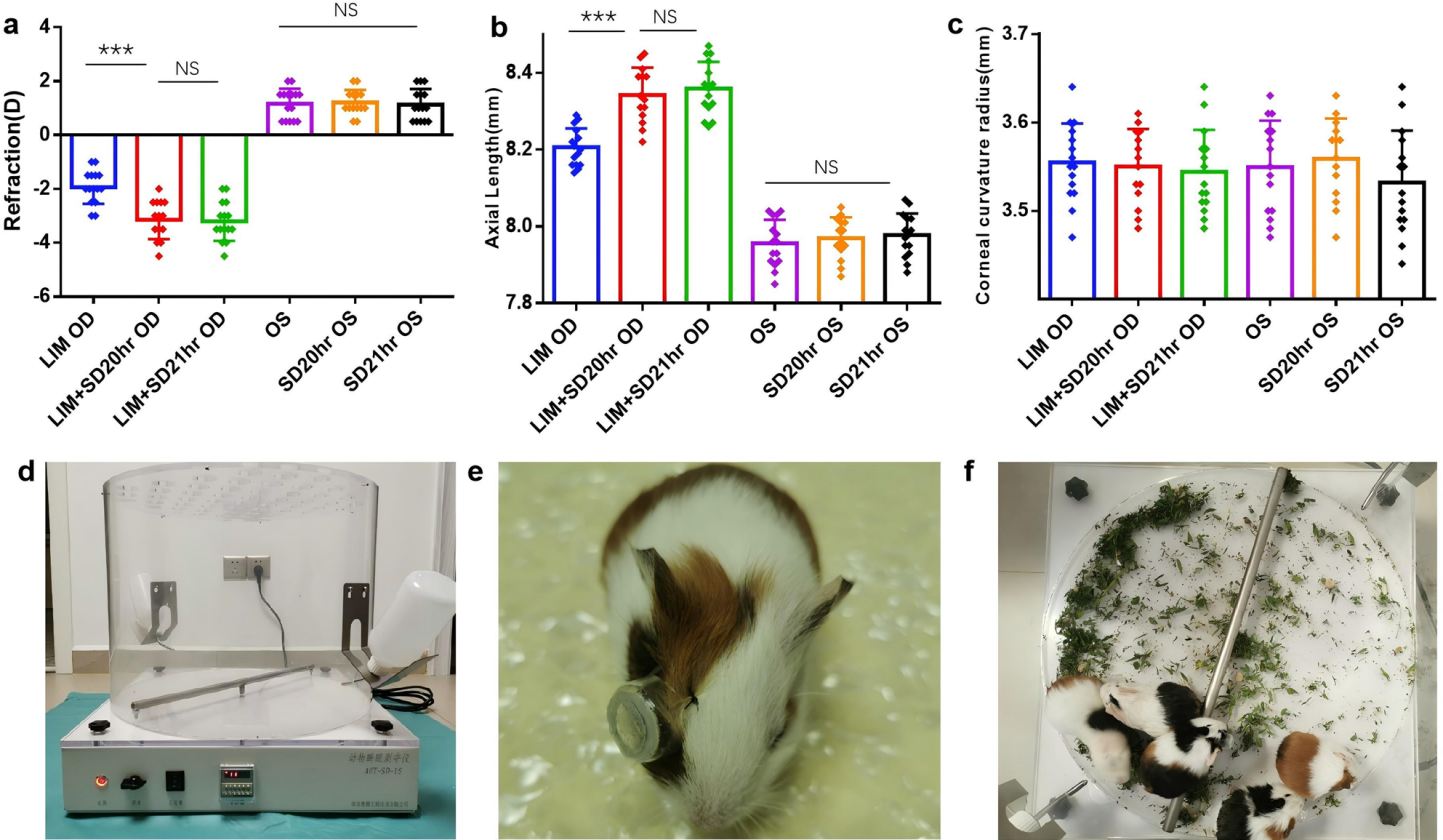
The various degrees of myopic progression are depicted in terms of refraction **(a)**, axial length **(b)**, and corneal curvature radius **(c)**. Panel (**d**) illustrates the instrument used for inducing sleep deprivation. Panel (**e**) shows a guinea pig with a lens sutured to its right eye. In panel (**f**), the animals are positioned on an automated random motion platform. LIM OD, lens-induced myopia in right eyes (*n* = 15); OS, left eye (*n* = 15); LIM+SD20hr OD, combination of lens-induced myopia and 20 h of sleep deprivation per day in right eyes (*n* = 14); SD20hr OS, 20 h of sleep deprivation per day in left eyes (*n* = 14); LIM+SD21hr OD, combination of lens-induced myopia and 21 h of sleep deprivation per day in right eyes (*n* = 15); SD21hr OS, 21 h of sleep deprivation per day in left eyes (*n* = 15). Statistical significance is indicated as follows: ***, *p* < 0.001; NS, no significant difference

Guinea pigs were housed in cages under a controlled 12-hour light/dark cycle, with illumination from 7:00 AM to 7:00 PM, for a duration of 28 consecutive days. The right eyes of all subjects were utilized for the induction of lens-induced myopia (LIM), employing a − 6.00 diopter lens that was sutured and secured to the periorbital soft tissue, as documented in previous studies [28] (Fig. 1e). The lenses were cleaned daily to maintain clarity. The time course of myopia induction was 2 weeks. The contralateral left eyes, which were not exposed to LIM but were subjected to the same systemic effects of SD, functioned as internal controls analogous to an SD-only condition. All measurements were performed on both eyes. The corneal curvature radius was evaluated using a keratometer, while refractive error, expressed in diopters, was determined using a computerized optometry unit following the administration of compounded tropicamide to induce cycloplegia. Axial length, defined as the distance from the corneal surface to the retinal pigment epithelium and measured with an accuracy of 0.01 mm, was assessed using ophthalmic A/B ultrasound. Each parameter was measured three times by the same operator, and the mean value was utilized for analysis.

This study involves two parts. Forty-five animals were used in the first part and were evenly divided into three groups: the LIM group, LIM+SD20hr group, and LIM+SD21hr group. Thirty-six animals were used in the second part and were divided into three groups: the LIM+SD20hr, LIM+SD20hr + NE-100, and LIM+SD20hr+saline group. A mouse sleep deprivation apparatus (BW-NSD404) (Fig. 1d) was employed, utilizing an automated random motion platform [29] over a period of 14 consecutive days. The platform operated at a rotational speed of three rotations per 10 min, delivering unpredictable gentle rotations to disrupt sleep posture without inducing undue physical exertion. Wakefulness was monitored through continuous video surveillance and periodic behavioral assessments for indicators of sleep, such as closed eyes and immobility exceeding 30 s. In the LIM+SD20hr group, animals were subjected to sleep deprivation for 20 h daily, during which they wore a concave lens on the right eye, and the motion platform was in rotation. This 20-hour period encompassed 12 h of light and 8 h of darkness. In contrast, animals in the LIM+SD21hr group experienced sleep deprivation for 21 h each day. For the LIM+SD20hr + NE-100 group, peribulbar injections of 60 µg NE-100, a S1R antagonist, were administered to the right eye, following previously established protocols [27]. Animals in the LIM+SD20hr+saline group received saline injections instead. Notably, one guinea pig from the LIM+SD20hr + NE-100 group and another from the LIM+SD20hr group did not survive the study.

### Administration method

The administration of NE-100 or saline commenced on the day of sleep deprivation. NE-100 was prepared in a saline solution at a concentration of 0.6 µg/µL. A topical application of 0.4% oxybuprocaine hydrochloride ophthalmic solution and 1% ropivacaine hydrochloride was applied to the right eye. Subsequently, in accordance with the methodology described by Jiang et al. [30], daily peribulbar injections of 100 µL NE-100 were administered using a 30-gauge needle for a duration of 14 consecutive days [27]. Peribulbar injections of saline were administered as a control.

### Immune histochemistry of S1R

Ocular specimens from the LIM, LIM+20 h, and LIM+21 h groups were utilized for retinal immunohistochemistry to detect the S1R protein. The guinea pigs were euthanized via intraperitoneal injection of an overdose of pentobarbital sodium (Nembutal; AKORN; Lake Forest, IL). Subsequently, their eyeballs were excised and fixed in 10% neutral buffered formaldehyde. The specimens were then embedded in paraffin and sectioned into 5 μm slices. The sections underwent deparaffinization using xylene and were rehydrated through a series of ethanol solutions, culminating in phosphate-buffered saline. The primary antibody employed was a rabbit anti-S1R polyclonal antibody at a dilution of 1:100. Immunohistochemistry was conducted using the Polink-2 plus Polymer HRP Detection System.

### Western blotting

Enucleated eyecups were dissected to carefully remove the anterior portion of eye tissues using a pair of tweezers, including the cornea, the iris, the lens and the ciliary body, reserve the posterior portion of eyecups. The retinal neuroepithelium layer was separated from the posterior portion of eyecups and was used for Western blot testing to detect the expression of retinal S1R protein in the LIM group (*n* = 4) and the LIM+20 h group (*n* = 4). Then, it was weighed and lysed with protein extraction lysis buffer, and underwent centrifugation at 9000 g at 4 °C for 15 min. The supernatant was collected and stored at − 20 °C. A bicinchoninic acid protein quantification kit was employed for protein quantification. A 25-µg sample of protein was separated by 10% discontinuous sodium dodecyl sulfate polyacrylamide gel electrophoresis. The protein was electro-transferred to a polyvinylidenfluoride membranes, which then was blocked with 5% skimmed milk. Rabbit anti-S1R polyclonal antibody was added (1:500) and preserved at 4 °C overnight. Horseradish peroxidase-labeled goat anti-rabbit IgG was incubated for 90 min post-elution. The chemiluminescent reagent was added to react for 5 min after the membrane was washed, after which it underwent x-ray exposure, image displaying, and photographic fixing. The Bandscan 5.0 image analysis software was used to analyze the gray level of the target strip, and GAPDH was used as the internal control to compute the relative expression of S1R protein (relative expression of S1R = gray level of S1R strip/gray level of GAPDH strip).

### High-performance liquid chromatography electrochemical measurement of Glu and GABA

The retinal neuroepithelium layer was utilized for high-performance liquid chromatography with electrochemical detection (HPLC-EC) to quantify the concentrations of Glu and GABA. Samples were collected from four experimental groups: LIM (*n* = 9), LIM+SD20hr (*n* = 12), LIM+SD20hr + NE-100 (*n* = 11), and LIM+SD20hr+saline (*n* = 11). The retinal neuroepithelium layer was excised, weighed, and preserved in liquid nitrogen. Chromatographic analysis was conducted using a Waters µBondapak C18 reverse-phase column (150 mm x 3.9 mm, 5 μm). The mobile phase consisted of an aqueous solution (containing 2.8 g of B8, 6.8 g of KH2PO4, and 18 mg of ethylenediaminetetraacetic acid per 500 mL, pH 3.3), methanol, and acetonitrile in a ratio of 78:19:3. The flow rate was maintained at 0.75 mL/min, with a working voltage of 0.7 V and an injection volume of 10 µL. The retention times for Glu and GABA were 2.9 and 6.2 min, respectively. Subsequently, the content of Glu and GABA was calculated in nanograms per milligram of the nerve retina.

### Statistical analysis

All data were presented as means ± standard deviations (SD) and analyzed using the SPSS 22.0 software package (IBM Corp., Armonk, NY). The normality of the data distribution for all variables was assessed using the Shapiro-Wilk test. The corneal radius of curvature, refractive error, axial length, and relative expression levels of S1R protein, Glu, and GABA in guinea pigs were examined using one-way ANOVA to assess differences among groups. For multiple comparisons between groups, a Student’s unpaired t-test with Bonferroni correction was employed, except for S1R expression, which was analyzed using the nonparametric Kruskal-Wallis test due to its non-normal distribution as determined by the Shapiro-Wilk test. *P* < 0.05 was considered indicative of statistical significance.

## Results

Following a two-week period of lens wear on the right eye, the LIM eyes exhibited a significant reduction in refraction (LIM OD: -1.93 ± 0.16 D vs. OS: 1.17 ± 0.14 D, *p* < 0.0001) (Fig. 1a) and an increase in axial length (LIM OD: 8.21 ± 0.01 mm vs. OS: 7.96 ± 0.02 mm, *p* < 0.0001) (Fig. 1b) compared to the left eyes. Considering that guinea pigs are typically awake for approximately 15 h per day (31), we employed automated random motion platforms to induce sleep deprivation for 20 h daily. The random movement of the standing platform effectively prevented the animals from falling asleep. Under conditions of 20-hour sleep deprivation, the LIM right eyes demonstrated an increase in refractive power (LIM+SD20hr OD: -3.14 ± 0.19 D vs. LIM OD: -1.93 ± 0.16 D, *p* < 0.0001) (Fig. 1a) and further elongation of axial length (LIM+SD20hr OD: 8.34 ± 0.02 mm vs. LIM OD: 8.21 ± 0.01 mm, *p* < 0.0001) (Fig. 1b) compared to LIM eyes without sleep deprivation. In contrast, after 20 h of sleep deprivation, the control left eyes exhibited no significant changes in refraction (OS: 1.17 ± 0.14 D vs. SD20hr OS: 1.21 ± 0.13 D, *p* = 0.81) or axial length (OS: 7.97 ± 0.01 mm vs. SD20hr OS: 7.96 ± 0.02 mm, *p* = 0.54). Subsequently, we aimed to investigate the impact of extended sleep deprivation on ocular biological parameters. Consequently, an additional experiment involving 21 h of sleep deprivation was conducted on a separate cohort of guinea pigs with LIM in the right eye. The results indicated no significant differences between the LIM+SD20hr group and the LIM+SD21hr group in terms of refraction (LIM+SD20hr OD: -3.14 ± 0.19 D vs. LIM+SD21hr OD: -3.20 ± 0.19 D, *p* = 0.83) and axial length (LIM+SD20hr OD: 8.34 ± 0.02 mm vs. LIM+SD21hr OD: 8.36 ± 0.02 mm, *N* = 15, *p* = 0.52). Additionally, no changes were observed in the corneal curvature radius across all groups (Fig. 1c).

**Fig. 2:**
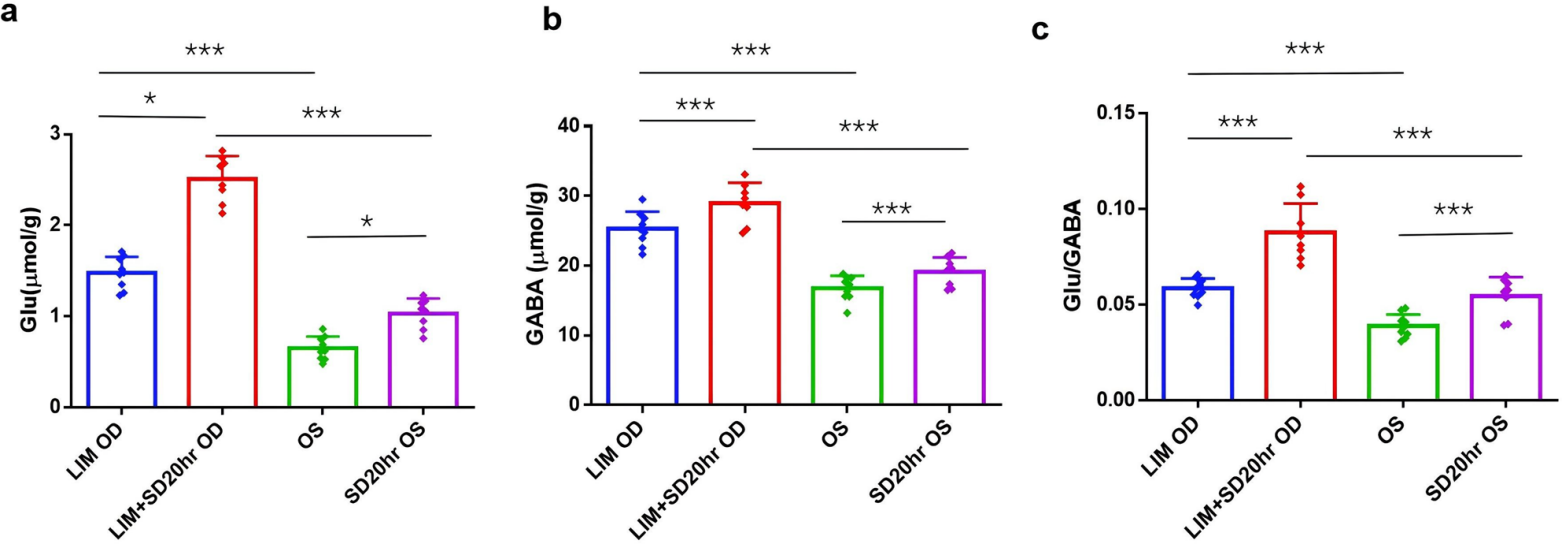
(**a**) The concentration of Glu (µmol/g) in the retina in 4 groups: LIM OD (*n* = 9), LIM+SD20hr OD (*n* = 8), OS (*n* = 9), SD20hr OS (*n* = 8). (**b**) The concentration of GABA (µmol/g) in the retina. (**c**) The ratio of Glu/GABA. Data are mean ± SD. LIM, lens-induced-myopia; OD, right eye; OS, left eye; SD, sleep deprivation; ***, *p* < 0.001; *, *p* < 0.05

In light of the role of SD in influencing refractive diopters and axial length in LIM eyes, we conducted an investigation into the impact of SD on myopia-associated signaling factors, specifically GABA and Glu. HPLC was employed to quantify the retinal concentrations of GABA and Glu. The concentration of Glu was significantly elevated in the LIM OD compared to OS (LIM OD: 1.48 ± 0.06 µg/mol vs. OS: 0.65 ± 0.04 µg/mol, *p* < 0.0001). Furthermore, 20 h of SD resulted in an increase in Glu levels in both the LIM OD (LIM OD: 1.48 ± 0.06 µg/mol vs. LIM+SD20hr OD: 2.51 ± 0.09 µg/mol, *p* < 0.001) and OS (OS: 0.65 ± 0.04 µg/mol vs. SD20hr OS: 1.03 ± 0.06 µg/mol, *p* < 0.05) (Fig. 2a). Similarly, the concentration of GABA was higher in LIM OD than in OS (LIM OD: 25.30 ± 0.82 µg/mol vs. OS: 16.78 ± 0.59 µg/mol, *p* < 0.001). Post 20 h of SD, GABA concentrations increased in both LIM OD (LIM OD: 25.30 ± 0.82 µg/mol vs. LIM+SD20hr OD: 28.97 ± 1.024 µg/mol, *p* < 0.001) and OS (OS: 16.78 ± 0.59 µg/mol vs. SD20hr OS: 19.12 ± 0.73 µg/mol, *p* < 0.001) (Fig. 2b). The Glu/GABA ratio, a known indicator of myopia progression [11], was significantly higher in LIM OD (LIM OD: 0.06 ± 0.002 vs. OS: 0.04 ± 0.002, *p* < 0.001), as well as in sleep-deprived LIM OD (LIM OD: 0.06 ± 0.002 vs. LIM+SD20hr OD: 0.088 ± 0.005, *p* < 0.001) and OS (OS: 0.04 ± 0.002 vs. SD20hr OS: 0.05 ± 0.003, *p* < 0.001) (Fig. 2c).

**Fig. 3:**
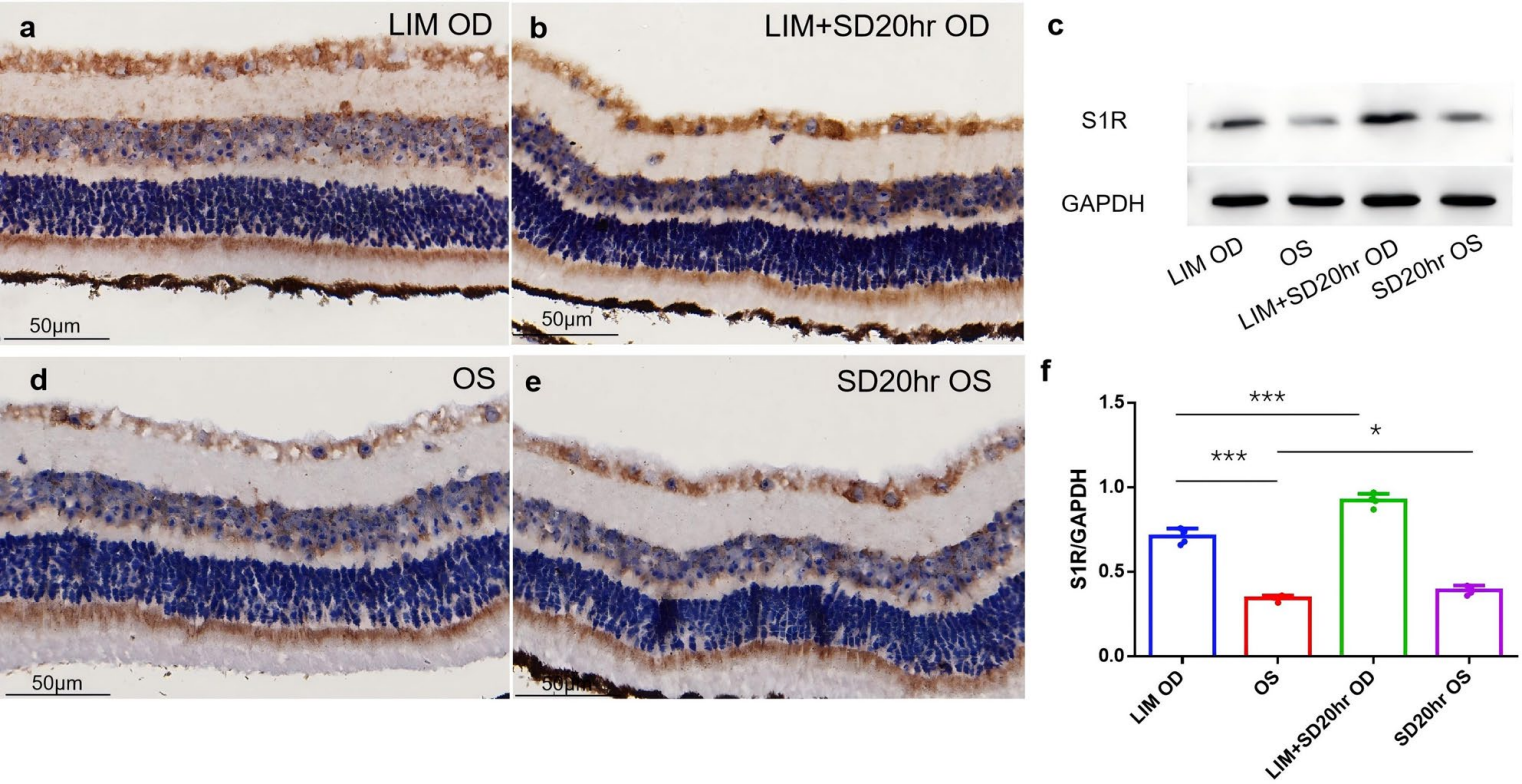
The immune histochemistry of S1R (brown staining) in LIM OD **(a)**, LIM+SD20hr OD **(b)**, OS **(d)**, and SD20hr OS **(e)**. (**c**) Western blot of S1R in the retinal of guinea pigs with GAPDH as a control (the full length blots are provided in supplementary files). (**f**) The expression of S1R relative to GAPDH. Data are mean ± SD (*n* = 4). LIM, lens-induced-myopia; OD, right eye; OS, left eye; SD, sleep deprivation; ***, *p* < 0.001; *, *p* < 0.05. OS is the contralateral control eye of OD

The immunohistochemical analysis of the retina reveals that S1R is predominantly expressed in the majority of retinal ganglion cells (RGCs), the inner segments of photoreceptors, and certain cells within the inner nuclear layer, as evidenced by faint brown staining (Fig. 3d). In SD20hr eyes there was a slight increase in S1R expression within the RGCs (Fig. 3e). In contrast, eyes subjected to LIM (Fig. 3a) and those subjected to both LIM and SD20hr (Fig. 3b) exhibited intensified S1R expression in the RGCs and the inner segments of photoreceptors. Additionally, there was an increase in S1R-positive cells within the inner and outer plexiform layers as well as the inner nuclear layer. Notably, a palisade arrangement of staining in Müller cells was observed in the LIM+SD20hr group. Further investigation of retinal S1R expression was conducted using Western blot analysis (Fig. 3c). The relative expression levels of S1R were determined to be 0.71 ± 0.02, 0.35 ± 0.01, 0.92 ± 0.02, and 0.39 ± 0.01 in LIM OD, OS, LIM+20 h OD, and SD20hr OS, respectively. S1R expression was significantly higher in LIM OD compared to OS (*p* < 0.001) (Fig. 3f). Following sleep deprivation, S1R expression was significantly elevated in both LIM OD+SD20hr (*p* < 0.001) and OS (*p* < 0.05) (Fig. 3f).

**Fig. 4:**
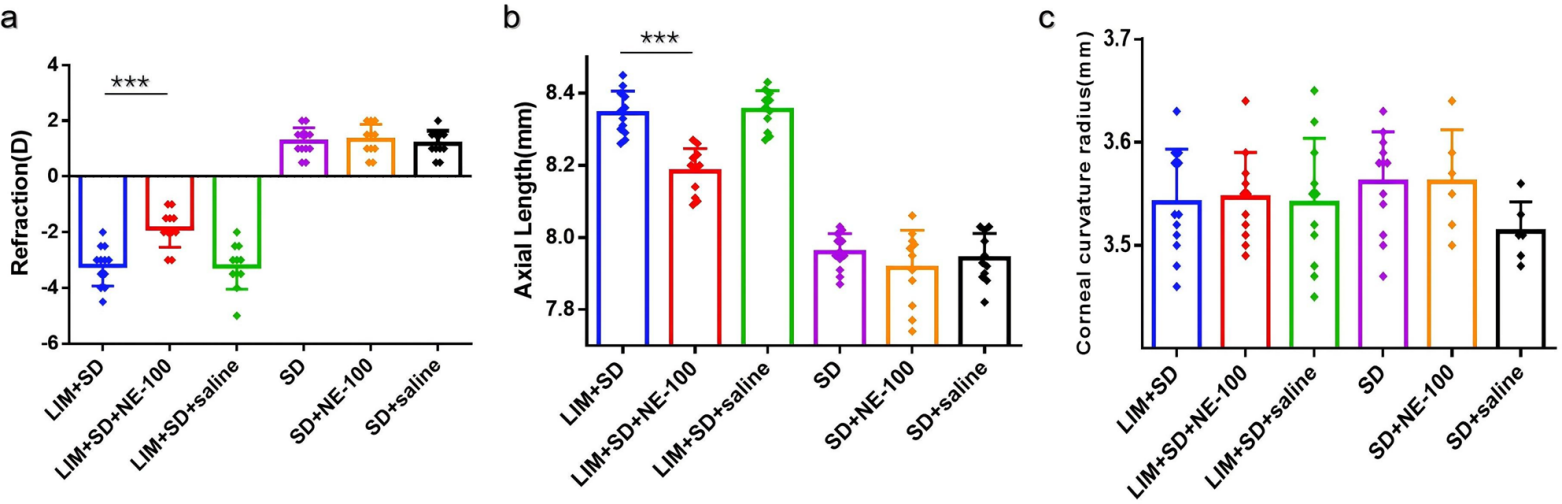
(**a**) The refractive status in 6 groups, (**b**) the axial length in 6 groups, (**c**) the corneal curvature radius in 6 groups. LIM + SD, sleep deprivation for 20 h daily combined with lens-induced-myopia (*n* = 12); LIM + SD+NE-100, sleep deprivation for 20 h daily combined with LIM and peribulbar injection of NE-100 (a S1R antagonist) (*n* = 11); LIM + SD+saline, sleep deprivation for 20 h daily combined with LIM and peribulbar injection of saline (*n* = 11); SD, sleep deprivation for 20 h daily (*n* = 12); SD + NE-100, sleep deprivation for 20 h daily combined with peribulbar injection of NE-100 (*n* = 11); SD+saline, sleep deprivation for 20 h daily combined with peribulbar injection of saline (*n* = 11); ***, *p* < 0.001

In response to the upregulation of S1R in the sleep-deprived eye, NE-100, a S1R antagonist, was administered to the sleep-deprived eye to assess its potential to reverse refractive changes. In the LIM eye, a two-week regimen of peribulbar NE-100 injections resulted in a significant increase in refractive power (LIM + SD+NE-100: -1.86 ± 0.20 D vs. LIM + SD: -3.21 ± 0.21 D, *p* < 0.001) (Fig. 4a) and a reduction in axial length compared to the LIM + SD group (LIM + SD+NE-100: 8.18 ± 0.02 mm vs. LIM + SD: 8.34 ± 0.02 mm, *p* < 0.001) (Fig. 4b). However, the corneal radius of curvature did not exhibit a significant difference between the two groups (LIM + SD+NE-100: 3.55 ± 0.01 mm vs. LIM + SD: 3.54 ± 0.01 mm, *p* = 0.82) (Fig. 4c). In eyes without LIM, NE-100 application did not affect ocular parameters, including refraction, axial length, and corneal radius of curvature. Furthermore, no significant differences were observed among the SD group, SD + NE-100 group, and SD+saline group.

**Fig. 5:**
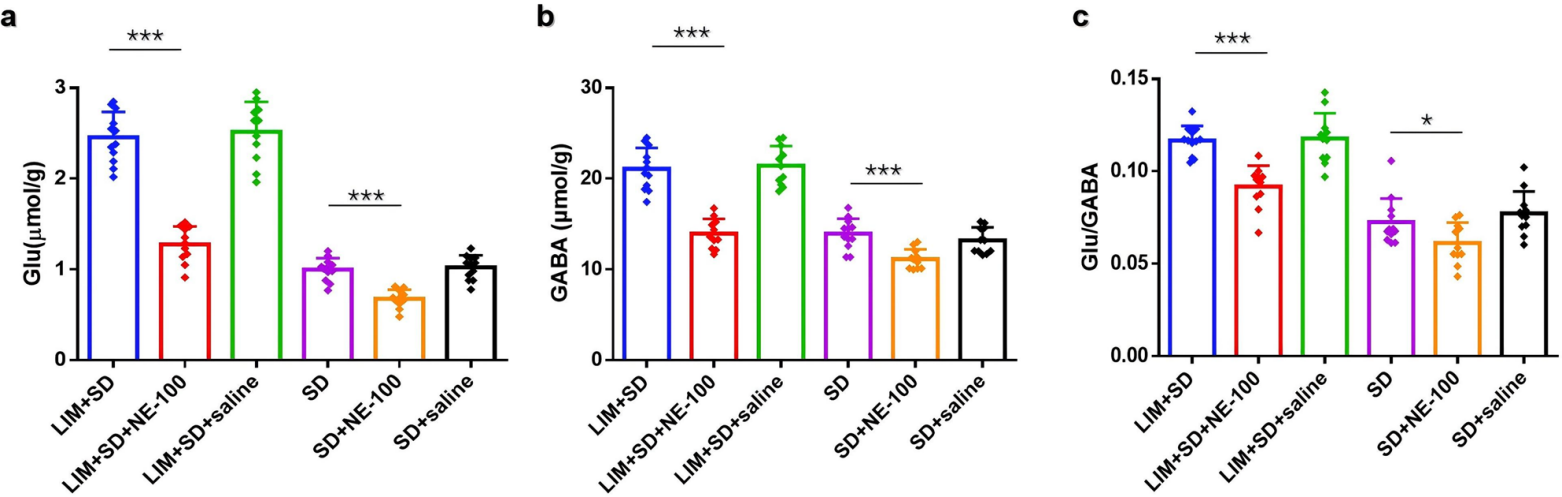
The varying concentrations of glutamate **(a)**, GABA **(b)**, and the Glu/GABA ratio in the eye **(c)**. The data are presented as mean ± SD. The experimental groups are as follows: LIM + SD, which involves 20 h of daily sleep deprivation combined with lens-induced myopia (*n* = 12); LIM + SD+NE-100, which includes 20 h of daily sleep deprivation combined with LIM and a peribulbar injection of NE-100 (*n* = 11); LIM + SD+saline, which consists of 20 h of daily sleep deprivation combined with LIM and a peribulbar injection of saline (*n* = 11); SD, which involves 20 h of daily sleep deprivation (*n* = 12); SD + NE-100, which includes 20 h of daily sleep deprivation combined with a peribulbar injection of NE-100 (*n* = 11); and SD+saline, which consists of 20 h of daily sleep deprivation combined with a peribulbar injection of saline (*n* = 11). Statistical significance is denoted as follows: ***, *p* < 0.001; *, *p* < 0.05

The results obtained from HPLC analyses were presented for the eyes. Administration of NE-100 led to a significant reduction in the retinal concentration of Glu (LIM + SD+NE-100: 1.28 ± 0.06 µmol/g compared to LIM + SD: 2.46 ± 0.08 µmol/g, *p* < 0.001) as depicted in Fig. 5a, GABA (LIM + SD+NE-100: 13.94 ± 0.49 µmol/g compared to LIM + SD: 21.08 ± 0.67 µmol/g, *p* < 0.001) as shown in Fig. 5b, and the Glu/GABA ratio (LIM + SD+NE-100: 0.09 ± 0.003 compared to LIM + SD: 0.12 ± 0.002 µmol/g, *p* < 0.001) as illustrated in Fig. 5c. Similarly, in eyes not subjected to LIM treatment, NE-100 also resulted in a decreased concentration of Glu (SD + NE-100: 0.68 ± 0.03 µmol/g compared to SD: 1.00 ± 0.04 µmol/g, *p* < 0.001) as shown in Fig. 5a, GABA (SD + NE-100: 11.14 ± 0.32 µmol/g compared to SD: 13.93 ± 0.48 µmol/g, *p* < 0.001) as depicted in Fig. 5b, and the Glu/GABA ratio (SD + NE-100: 0.06 ± 0.003 compared to SD: 0.07 ± 0.004, *p* < 0.05) as presented in Fig. 5c.

## Discussion

The relationship between SD and myopia remains inadequately understood. Neurobiological research has demonstrated that SD can influence signal transmission mediated by neurotransmitters such as Glu and GABA [31]. Activation of the S1R has been shown to exert a regulatory effect on these neurotransmitters [26]. Investigations using animal models of myopia have confirmed the involvement of retinal Glu and GABA in the regulatory processes underlying myopia development [11]. Consequently, we hypothesize that SD may regulate retinal S1R and its downstream signaling pathways by altering myopia-related signaling factors within the retina, thereby influencing myopia formation. Previous studies have identified LIM in guinea pigs as a well-established model for examining the pathogenesis of myopia [32]. Additionally, S1R is a crucial protein initially cloned in guinea pigs and is expressed in the endoplasmic reticulum of Müller cells and ganglion cells [18]. Therefore, in this study, we developed a novel guinea pig LIM + SD model, building upon the existing guinea pig LIM model, and conducted a series of investigations.

During the growth and development of the eye, maintaining a regular circadian rhythm is crucial for achieving emmetropization [33]. In a typical visual environment, the ocular axial extension exhibits significant circadian rhythm variations, peaking around midday [33]. In cases of animal models and human myopia, disruptions in the normal circadian rhythm of axial length changes lead to accelerated extension at night, surpassing the rate of emmetropization and ultimately resulting in myopia [34]. SD may lead to cognitive impairment and circadian dysfunction in rat models [35]. The current commonly used SD models include rapid eye movement (REM) SD and total SD; the former deprives of REM sleep phase only and is primarily related to dreaming and depression. The latter deprives the entire sleep cycle [36]. The common lack of sleep among adolescents is the shortening of overall sleep time and the decline of sleep quality [37], which the total SD model could reflect. The SD instrument in this study uses the random movement of a standing platform for experimental animals to prevent them from falling asleep. For this instrument, the exercise and rest time can be modified. Besides, it does no physical harm to the animals. The wakefulness of guinea pigs is evenly distributed in the day and night, and they normally awake for approximately 15 h a day [38]. The findings of our study indicated that a period of 20 h of SD can result in alterations in the refractive status of guinea pigs. In order to investigate the potential exacerbating effects of prolonged sleep deprivation on refractive state alterations, we extended the period of sleep deprivation to 21 h. However, our analysis of ophthalmic biological parameters in guinea pigs did not reveal statistically significant differences. Hence, we applied 20 h of SD for the subsequent experiments.

Emmetropia relies on the accurate alignment of eye measurements such as axial length and corneal curvature radius, any imbalance can cause refractive errors like myopia [39]. Studies have found that among children and adolescents, compared to emmetropic group, the myopic group showed increased axial length and decreased corneal curvature radius [40]. The findings of this study indicate that, in comparison to the control eye, the increased diopters and elongated axial length observed in the LIM eyes confirm the successful establishment of the LIM model. Within this study, the contralateral left eyes in the LIM + SD groups served effectively as internal controls for SD-only conditions. These eyes were not subjected to optical defocus but experienced the same systemic effects of SD, such as stress and metabolic changes, as the right eyes. Our measurements revealed no significant changes in refractive error or axial length in these left eyes compared to baseline or non-SD conditions, suggesting that, under the current experimental conditions, SD alone was not associated with detectable myopic shifts in the absence of defocus. Conversely, the right eyes demonstrated exacerbated myopia progression, indicating a synergistic interaction between SD and LIM rather than an independent effect of SD alone under the present paradigm. This within-animal paired design enhances control over confounding variables and corroborates previous studies that have shown circadian disruptions can exacerbate myopia under environmental stimuli [1]. Subsequent observations of intraocular concentrations of myopia-related factors indicated that (SD could elevate levels of GABA, Glu, and the Glu/GABA ratio in the retina. We hypothesize that alterations in Glu and GABA levels enhance the sensitivity of the eye to the optical defocus signal induced by negative lenses, thereby facilitating the progression of LIM. Our findings align with human research linking shorter sleep to higher myopia risk. A meta-analysis found that less sleep in children and adolescents increases myopia risk, while more sleep may protect against it [41]. Similarly, a study of Chinese students showed that sleeping over 10 h daily reduced myopia risk compared to less than 8 h, possibly due to physical activity [42]. These findings suggest sleep deprivation may worsen myopia through shared mechanisms like neurotransmitter issues, highlighting the importance of sleep interventions in preventing myopia.

The association between SD and myopia remains a contentious topic within the academic community. A cross-sectional study conducted among nine-year-old students in Singapore found no independent relationship between sleep duration and quality and the incidence of myopia [43]. Similarly, a longitudinal study primarily involving Caucasian participants, which followed sleep behavior from childhood to young adulthood over a 12-year period, reported no significant association between sleep and myopia [44]. In contrast, a study conducted in China among students aged 6 to 18 years identified sleep durations of less than 7 h as a risk factor for both myopia and high myopia, as determined by univariate analysis [45]. Based on our findings, under the current experimental conditions, we suggest that SD may not play a critical role in the initial onset of myopia. Instead, it appears to accelerate the progression of myopia when an optical defocus signal is present. Sleep deprivation alone was not associated with detectable myopic changes in this model; however, it may hasten the development of myopia in individuals already affected by the condition. Consequently, the impact of SD on myopia progression may vary when a larger cohort of individuals with myopic status is considered.

We previously found that the expression of S1R was elevated in form deprivation myopia induced in guinea pigs and the refraction power was inhibited by applying NE-100 at a concentration of 60 µg [27]. In this study, we observed activation of the S1R following the establishment of LIM in guinea pigs, with its expression further increasing under conditions of SD. The involvement of S1R in the development of myopia was corroborated through the use of the antagonist NE-100, which effectively inhibited the progression of myopic refractive error and axial elongation, although it did not completely restore these parameters to the levels observed in the contralateral control eyes. Notably, no such changes were detected in the contralateral eyes subjected to SD alone under the current experimental conditions. This phenomenon may be attributed to the activation of myopia-associated signaling pathways involving S1R, Glu, and GABA, both of which were reduced following NE-100 administration. Despite this, the S1R antagonist only partially mitigated changes in refractive status and axial length, indicating the presence of additional factors contributing to myopia development. In conclusion, this research identifies a novel link between SD and the progression of myopia in subjects with preexisting myopia, highlighting S1R as a potential mediator. This finding opens new avenues for further research into preventive and therapeutic strategies for myopia, as well as for understanding the molecular mechanisms underlying myopic progression.

A limitation of this study is the lack of direct measurements of stress hormones, such as plasma corticosterone, which may be elevated due to SD and could potentially influence scleral remodeling or neurotransmitter metabolism [46]. Although we cannot entirely rule out these effects, our findings suggest that SD acts synergistically with LIM rather than independently driving refractive changes in this model. This is corroborated by the absence of significant myopic shifts in the contralateral left eyes under the current experimental conditions, which were exposed to the same systemic stress but did not undergo optical defocus. Furthermore, the partial reversal of effects by the S1R antagonist NE-100 indicates a specific mechanistic pathway beyond general stress responses, consistent with studies linking sleep disruptions to altered neuroplasticity and the exacerbation of myopia under environmental stimuli [31]. Nonetheless, future investigations should include stress biomarker assays such as corticosterone levels to definitively disentangle these confounds and enhance mechanistic clarity.

## Conclusions

Based on the aforementioned results, we conclude that SD is associated with the promotion of myopia development in LIM eyes. The induction of myopia may be partially inhibited by the S1R antagonist NE-100, suggesting that the S1R may play an associative role in myopia regulation under SD conditions. However, the underlying mechanisms involved in myopia control warrant further investigation.

## Supplementary Information

Below is the link to the electronic supplementary material.

Supplementary Material 1

Supplementary Material 2

## Data Availability

All data generated or analyzed during this study are included in this article.

## Abbreviations

DA: Dopamine
D2R: Dopamine D2 receptor
DAT: Dopamine transporter
EDTA: Ethylenediaminetetraacetic acid
GABA: γ-Aminobutyric acid
Glu: Glutamtic acid
HPLC-EC: High-performance liquid chromatography electrochemical
LIM: Lens-induced myopia
NE-100 N: N-diethyl-2-[4-methoxy-3-(2-phenylethoxy) phenyl] ethanamine hydrochloride
NO: Nitric oxide
NS: No significant difference
OD: Right eye
OS: Left eye
PVDF: Polyvinylidenfluoride
RA: Retinoic acid
REM: Rapid eye movement
RGC: Retinal ganglion cell
SD: Sleep deprivation
SDS: Sodium dodecyl sulfate
VIP: Vasoactive intestinal peptide
