# Supplementary figures and images for: The effect of sleep deprivation on lens-induced-myopia in guinea pigs
Source: Lab Anim Res. 2026 Jun 25;42:22. doi: 10.1186/s42826-026-00285-7 (PMC13295380; doi:10.1186/s42826-026-00285-7)

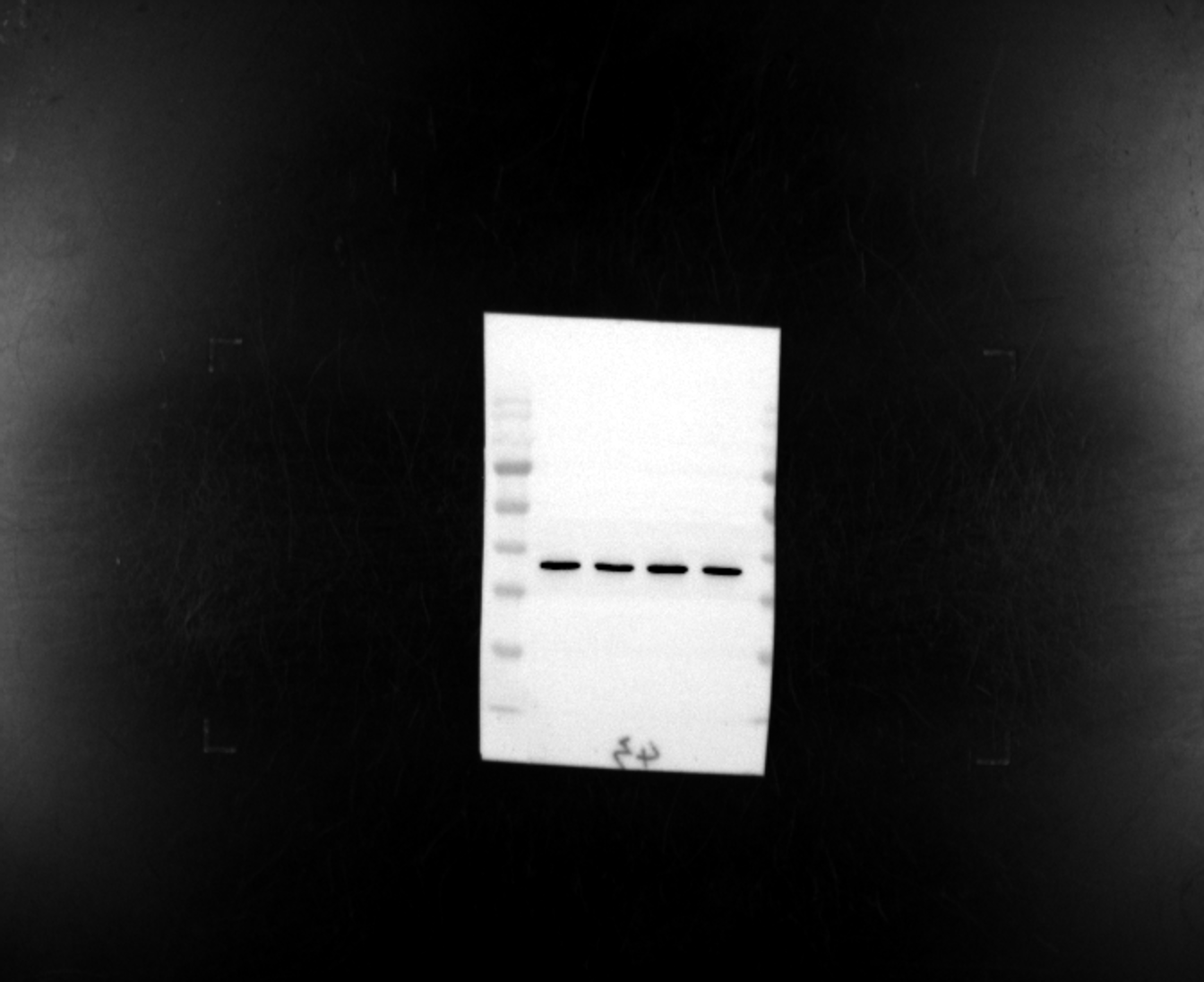

Supplement: Supplementary file 1 — Supplementary Material 1 [file 42826_2026_285_MOESM1_ESM.tif]

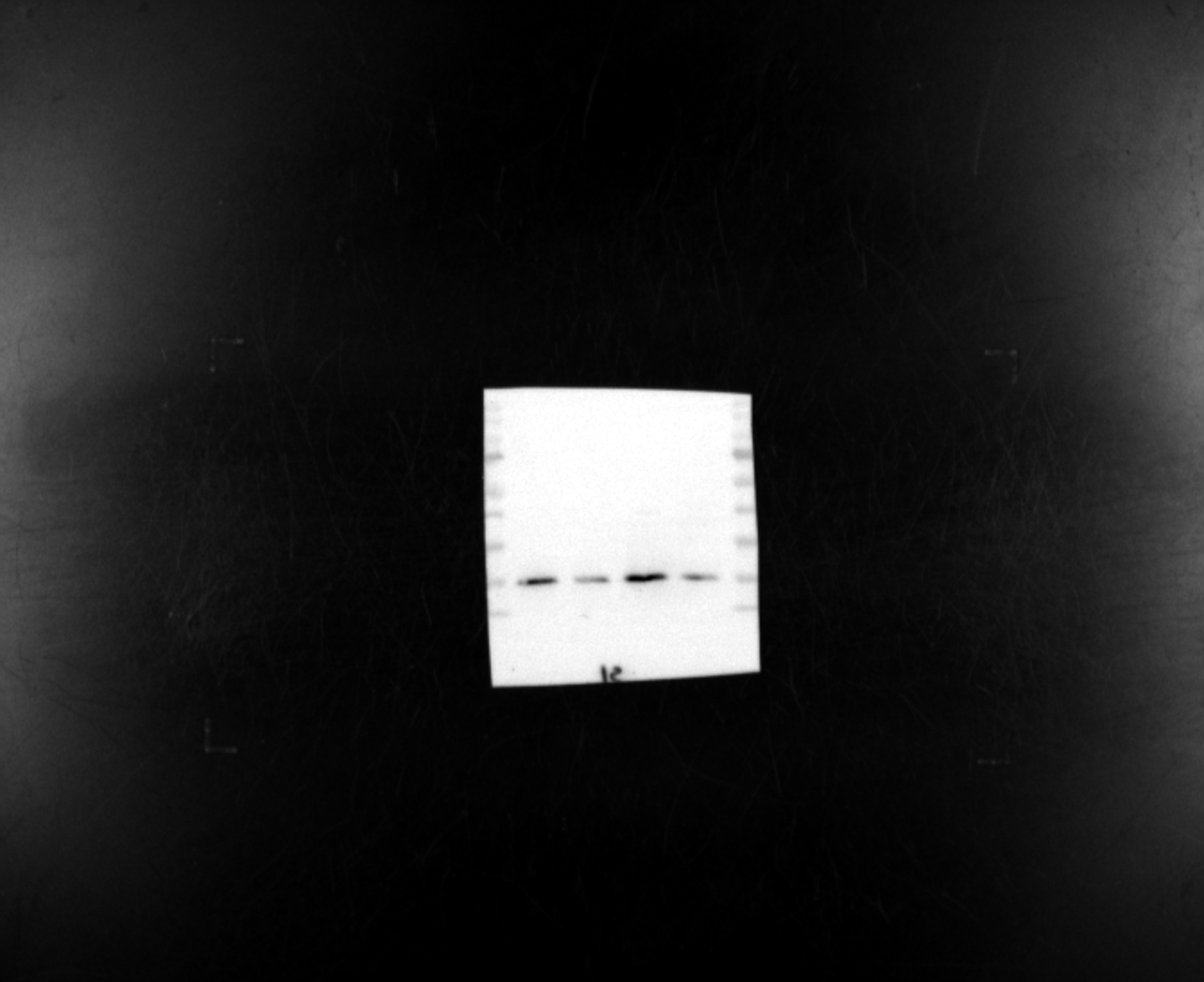

Supplement: Supplementary file 2 — Supplementary Material 2 [file 42826_2026_285_MOESM2_ESM.tif]
